# Constitutive Activity in an Ancestral Form of Abl Tyrosine Kinase

**DOI:** 10.1371/journal.pone.0131062

**Published:** 2015-06-19

**Authors:** Saadat U. Aleem, Barbara P. Craddock, W. Todd Miller

**Affiliations:** Department of Physiology and Biophysics, Stony Brook University, Stony Brook, NY, 11794, United States of America; University of Georgia, UNITED STATES

## Abstract

The c-*abl* proto-oncogene encodes a nonreceptor tyrosine kinase that is found in all metazoans, and is ubiquitously expressed in mammalian tissues. The Abl tyrosine kinase plays important roles in the regulation of mammalian cell physiology. Abl-like kinases have been identified in the genomes of unicellular choanoflagellates, the closest relatives to the Metazoa, and in related unicellular organisms. Here, we have carried out the first characterization of a premetazoan Abl kinase, MbAbl2, from the choanoflagellate *Monosiga brevicollis*. The enzyme possesses SH3, SH2, and kinase domains in a similar arrangement to its mammalian counterparts, and is an active tyrosine kinase. MbAbl2 lacks the N-terminal myristoylation and cap sequences that are critical regulators of mammalian Abl kinase activity, and we show that MbAbl2 is constitutively active. When expressed in mammalian cells, MbAbl2 strongly phosphorylates cellular proteins on tyrosine, and transforms cells much more potently than mammalian Abl kinase. Thus, MbAbl2 appears to lack the autoinhibitory mechanism that tightly constrains the activity of mammalian Abl kinases, suggesting that this regulatory apparatus arose more recently in metazoan evolution.

## Introduction

Mammalian Abl nonreceptor tyrosine kinases play important roles in signaling pathways that regulate actin binding and remodeling, cell adhesion and motility, and the DNA damage response (reviewed in [1]). As is generally true for mammalian tyrosine kinases, the activity of the normal cellular form of Abl is strictly regulated [1,2]. In patients with chronic myelogenous leukemia (CML), a chromosomal translocation results in the production of a constitutively active Bcr-Abl fusion protein [3,4]. The high tyrosine kinase activity of Bcr-Abl triggers the uncontrolled proliferation of hematopoietic cells in CML [3,5].

**Fig 1:**
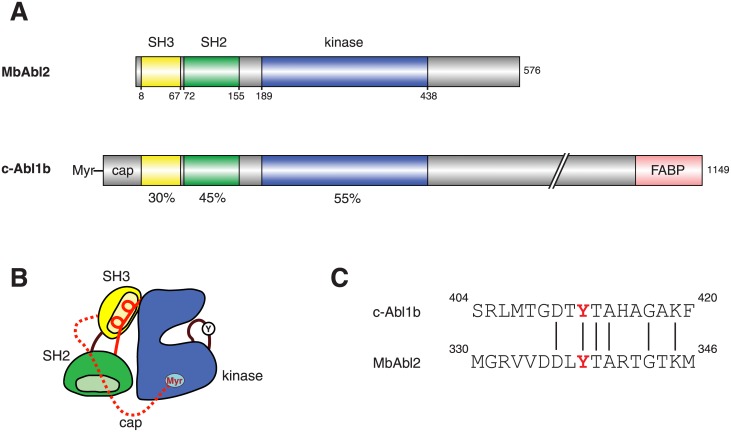
Domain organization of MbAbl2. (A) A schematic diagram showing the domain organization of MbAbl2 and mammalian Abl (murine c-Abl, 1b isoform). The percent amino acid identity for each domain is shown below the figure. (B) Cartoon representing the autoinhibited structure of mammalian Abl. The SH3 domain is in yellow, the SH2 domain is green, and the kinase domain is blue. The N-terminal myristoyl group (Myr) binds in a pocket on the kinase domain. The cap sequence is shown by a dashed red line, and the intramolecular SH3 ligand is shown by a solid red line. Tyrosine 412 (Y) in the activation loop is pictured. (C) Sequence comparison at the autophosphorylation site within the kinase activation loop.

In vertebrates, there are two closely related Abl proteins (Abl1 and Abl2) with similar domain architectures [1]. The N-terminal portion of Abl contains SH3, SH2, and tyrosine kinase domains in an arrangement that is reminiscent of Src-family kinases (Fig 1a). As in Src, the SH3 and SH2 domains dock onto the kinase catalytic domain and stabilize an autoinhibited conformation [2]. In Abl, an N-terminal myristoyl group binds to a pocket in the kinase domain, and an N-terminal cap region makes additional contacts that inhibit catalytic activity [2,6]. The large C-terminal portion of Abl contains multiple functional domains, including polyproline sequences and DNA- and actin-binding regions [1].

The evolutionary origin of Abl tyrosine kinase predates the split between multicellular animals and their unicellular ancestors. In nonvertebrate metazoans, including *C*. *elegans* and *D*. *melanogaster*, the arrangement of domains is very similar to that observed in mammals [1]. Abl genes have been identified in the unicellular choanoflagellate *M*. *brevicollis* [7–9], as well as in the filastereans *C*. *owczarzaki* and *M*. *vibrans* [10] and three ichthyosporean species [11]. In each of these premetazoan Abl kinases, the SH3-SH2-kinase domain architecture is preserved, but the large C-terminal portion is absent. The presence of the SH3-SH2-kinase domains indicates that this architecture was established before the divergence of filastereans from the choanoflagellate and metazoan clades. Furthermore, the four major nonreceptor tyrosine kinase families that contain this arrangement of domains (Src, Abl, Csk, and Tec) had already diverged from one another in premetazoan kinomes. In mammalian tyrosine kinases, this domain arrangement is critical for autoinhibition as well as for substrate recognition [9,12–15].

The Src kinases from the choanoflagellates *M*. *ovata* and *M*. *brevicollis*, and from the filastereans *C*. *owczarzaki* and *M*. *vibrans*, lack the tight regulation that is observed in mammalian Src kinases. For *M*. *ovata* and *M*. *brevicollis*, a C-terminal Src kinase (Csk) is present that phosphorylates the Src C-terminus, but this modification does not result in the same degree of inhibition that is seen in mammalian Csk-Src pairs [16,17]. The Csk kinases in *C*. *owczarzaki* and *M*. *vibrans* lack catalytic activity, and the Src kinases have high basal activity [18,19]. The activities of premetazoan forms of Abl, however, have not been studied. The genome of *M*. *brevicollis* encodes two putative Abl family kinases (MbAbl1 and MbAbl2), both of which contain the conserved SH3-SH2-kinase domain structure [7]. MbAbl2 is of particular interest for studies of the evolution of kinase regulation, as it lacks the N-terminal myristoylation sequence and cap region. In this study, we have cloned and characterized *M*. *brevicollis* MbAbl2. While it possesses similar basic enzymatic properties as mammalian c-Abl, we show that it has increased cellular activity and transforming ability. The results are consistent with a model in which tight regulation of Abl kinases arose after the transition to multicellularity.

## Materials and Methods

### Materials

Plasmid pBMN-I-GFP (developed by Gary Nolan) was from Addgene; anti-phosphotyrosine antibody 4G10 was purchased from Millipore; chloroquine, polybrene, leupeptin, aprotinin, PMSF and anti-Flag-HRP were purchased from Sigma; PBS, DMEM and antibiotics were from Mediatech; fetal bovine serum was from VWR; low melting point agarose was from Lonza.

### Cloning of MbAbl2 and murine c-Abl

The full-length MbAbl2 cDNA was amplified by PCR from a *Monosiga brevicollis* cDNA library [20] using the 5’ primer GCCACCATGAGCGCGACCAAGAAGAACGACATTTTTGTG and the 3’ primer CTAAAGAAGACGCGAGGCCTTGTTGAGTTCTCG. The PCR product was ligated into the vector pCR-BluntII-TOPO using the Zero Blunt TOPO cloning kit (Invitrogen). The sequence and direction of the MbAbl2 insert was confirmed by DNA sequencing. The MbAbl2 insert was subsequently cloned into the BamHI and EcoRI sites of retroviral vector pBMN-I-GFP. A Kozak sequence was included 5’ of the coding sequence, and a Flag sequence was added 3’ of the coding sequence. For baculovirus expression of MbAbl2, the cDNA encoding residues 1–457 was amplified by PCR and subcloned into the BamHI and NotI sites of plasmid pFastbac-Htb. This construct encodes MbAbl2 with a His-tag at the N-terminus.

Murine c-Abl cDNA (encoding residues 1–660) was amplified by PCR and cloned into the BamHI and EcoRI sites of pBMN-I-GFP using the 5’ primer CGGGATCCGCCACCATGGGGCAGCAGCCTGGAAAAGTTCTTG and the 3’ primer GGAATTCGAGTTCCCTGCTGTCATCCTCACTAGCCCCTCTG. Kozak (5’) and Flag (3’) sequences were included in the construct. All constructs were verified by DNA sequencing.

### Insect cell expression and purification of MbAbl2

The pFastbac-MbAbl2 plasmid was used to transfect *Spodoptera frugiperda* (Sf9) cells using the Bac-to-Bac system (Invitrogen). After isolation of recombinant baculovirus and 2 additional rounds of amplification, the MbAbl2 virus was used to infect 600 ml of Sf9 cells at 0.8 x 10^6^ cells/ml. After 3 days, infected cells were harvested and lysed in a French pressure cell in 30 ml buffer containing 50 mM Tris-HCl (pH 8.5), 100 mM NaCl, 1% NP-40, 5 mM 2-mercaptoethanol, 10 μg/ml leupeptin, 10 μg/ml aprotinin, and 1 mM PMSF. Cell lysate was centrifuged at 10,000 rpm for 10 minutes, filtered using a 0.8 μm filter, and applied to a 3 ml Ni-NTA column (Qiagen). The column was washed with 80 ml of buffer containing 20 mM Tris-HCl (pH 8.5), 500 mM NaCl, 10% glycerol, 20 mm imidazole, and 5 mM 2-mercaptoethanol. MbAbl2 was eluted from the column using the same buffer containing 100 mM imidazole. Column fractions were analyzed by SDS-PAGE and by kinase assay, and fractions containing MbAbl2 were stored at -80°C.

### Kinase assays

Two *in vitro* kinase assays were used for MbAbl2. The activity of MbAbl2 towards various synthetic peptide substrates was measured using the phosphocellulose paper binding assay [21]. Reaction mixtures contained 30 mM Tris-HCl (pH 7.5), 20 mM MgCl_2_, 1 mg/ml bovine serum albumin, 0.4 mM ATP, [γ-^32^P]-ATP (30–50 cpm/pmol), and 0.5 mM peptide. The peptides used were: Src peptide, AEEEEIYGEFEAKKKKG; EGFR peptide, AEEEEYFELVAKKKG; Abl peptide, EAIYAAPFAKKKG; IR peptide, KKEEEEYMMMMG; and PKA peptide, LRRASLG [22–24].

Further kinetic experiments on MbAbl2 were performed using a continuous spectrophotometric assay [25,26]. In this assay, the production of ADP was coupled to the oxidation of NADH measured as a reduction in absorbance at 340 nm. Reactions were performed at 30°C in a final volume of 75 μl. The reactions contained 100 mM Tris pH 7.5, 10 mM MgCl_2_, 1 mM ATP, 1 mM phosphoenolpyruvate (PEP), 184 units/ml pyruvate kinase, 128 units/ml lactate dehydrogenase, and 0.6 mg/ml NADH.

### Generation of Abl- and MbAbl2-expressing cell lines

Phoenix-Eco packaging cells, human embryonic kidney 293T cells, and mouse NIH3T3 cells (ATCC) were maintained in complete media containing Dulbecco’s Modified Eagle’s Medium (Mediatech, Inc.), 10% heat inactivated FBS, 1000 IU/ml penicillin, 1000 IU/ml streptomycin, and 25 ng/ml amphotericin. Stable cells co-expressing GFP and either Flag-Abl or Flag-MbAbl2 were made using the Phoenix-Eco Cell Retrovirus packaging system. Briefly, sub-confluent Phoenix-Eco cells were transfected with pBMN-I-GFP vector control, pBMN-Abl or pBMN-MbAbl-2 together with helper plasmid (3:1) in the presence of 25μM chloroquine and X-tremeGENE (Roche). After 24 hours at 37°C, 5% CO_2_, the transfection media was replaced with complete media and the cells were incubated at 32°C 5% CO_2_ for 48 hours during which time the retrovirus was collected twice, 0.45 um filtered, then frozen at -80°C or used immediately. The retrovirus was added to 90% confluent NIH3T3 cells in the presence of 4ug/ml polybrene and incubated at 37°C, 5% CO_2_. The retroviral mixture was replaced with complete media after 24 hours. The cells were monitored for GFP expression using fluorescent microscopy with the brightest GFP expressing cells being further selected by FACS and expanded for use in subsequent assays.

### SDS PAGE and Western Blotting

NIH3T3 cells stably expressing Abl or MbAbl2 were grown to confluency in 10cm tissue culture dishes. Cells were washed in 4°C PBS and lysed in radioimmune precipitation assay buffer (50 mM Tris-HCl, pH 7.4, 150 mM NaCl, 5 mM EDTA, 1%sodium deoxycholate, 1% Nonidet (P-40) supplemented with the protease inhibitors leupeptin (10 μg/ml), aprotinin (10 μg/ml), and the phosphatase inhibitor Na_3_VO_4_ (0.2 mM). After incubating at 4°C for 1 hour, the lysates were clarified by high speed centrifugation. 100 μg samples were separated on 10% SDS polyacrylamide gels, and transferred onto PVDF membranes. Tyrosine phosphorylation of the cell lysates was determined using the anti-phosphotyrosine antibody 4G10 (Millipore) while expression of the proteins was confirmed with anti-Flag-HRP (Sigma).

### Colony Formation Assay

Low melting point agarose (750 μl of 0.5%) prepared in PBS (Mediatech) was added to wells of a sterile 12 well plate and allowed to solidify. 1000 cells were resuspended in 750 μl 0.375% agarose prepared in complete media and added to each well. After incubating at 37°C, 5% CO_2_ for 24 hours, 750 μl complete media was added to each well. The plate was incubated at 37°C 5% CO_2_ and the media was replaced twice weekly. After 28 days of incubation, the wells were fixed with methanol:acetic acid:water (1:1:8), stained with 0.01% crystal violet in 25% methanol and the colonies in each well were counted.

### Anchorage-independent Growth Assay

20,000 cells were resuspended in complete media and added to each well of a 24 well low attachment plate (Corning). After 6 days at 37°C, 5% CO_2_, the cells were removed from the wells, centrifuged and trypsinized for 5 minutes in 100 μl 0.25% trypsin/EDTA to ensure a single cell mixture. The cells were counted using a hemocytometer.

## Results

We used PCR to amplify the cDNA encoding MbAbl2 from an *M*. *brevicollis* cDNA library. The MbAbl2 gene encodes a 576-residue protein with predicted SH3, SH2, and kinase domains, which exhibit 30%, 45%, and 55% identity with mouse c-Abl1b, respectively (Fig 1A). MbAbl2 lacks an N-terminal myristoylation sequence and cap region. In mammalian Abl kinases, the myristate moiety binds to a pocket in the catalytic domain, and together with the cap produces an autoinhibited conformation [2] (Fig 1B). The C-terminal portion of MbAbl2 kinase is much smaller than that of metazoan Abl kinases, and the F-actin binding domain is missing (Fig 1A). In mammalian Abl kinases, autophosphorylation of a tyrosine residue within the kinase activation loop increases catalytic activity (Tyr412; murine c-Abl numbering is used throughout this paper) [1,27]; a tyrosine is conserved at that position in MbAbl2 (Fig 1C). The overall percent amino acid identity for the SH3-SH2-kinase domains is 36.4%; a sequence comparison of these domains is shown in S1 Fig.

**Fig 2:**
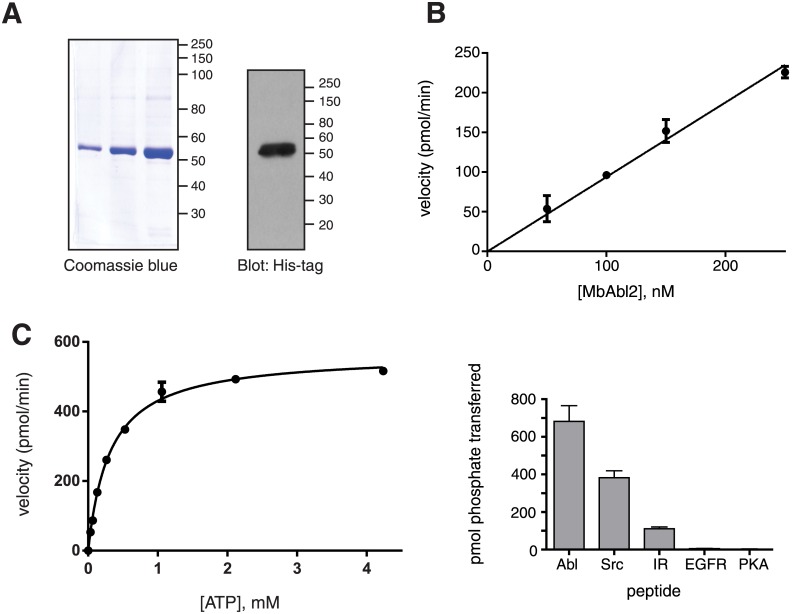
Enzymatic activity of MbAbl2. (A) SDS-PAGE analysis of purified MbAbl2, detected by Coomassie blue staining (left) or by anti-His-tag Western blotting (right). (B) Initial rates of substrate phosphorylation for various concentrations of MbAbl2 were measured in triplicate by the continuous spectrophotometric assay. The peptide substrate used, Abl peptide (EAIYAAPFAKKKG), was at a concentration of 100 μM. Error bars show standard deviations. (C) MbAbl2 initial rates were measured at varying concentrations of ATP using the continuous spectrophotometric assay. The fit to a hyperbolic substrate vs. velocity curve is shown. (D) MbAbl2-catalyzed phosphorylation of peptide substrates containing recognition motifs for various kinases. The peptide sequences are given in the Materials and Methods section. Enzymatic activity was measured using the phosphocellulose paper binding assay, with an enzyme concentration of 275 nM and peptide concentrations of 500 μM. Reactions proceeded for 10 minutes at 30°C. Error bars show standard deviation.

We expressed His-tagged MbAbl2 in Sf9 insect cells using a baculovirus vector and purified the protein using nickel-nitrilotriacetic acid resin (Fig 2A). MbAbl2 was an active tyrosine kinase, as measured toward a synthetic peptide substrate, and showed the expected linear relationship between enzyme concentration and velocity (Fig 2B). The turnover number of MbAbl2, 22 min^-1^, was higher than reported values for c-Abl (k_cat_ = 2.0 min^-1^) [28] or for the related Abl2 kinase (0.1 min^-1^) [29]. We note that MbAbl2 was assayed with an intact His-tag (as was Abl2), which could potentially affect k_cat_ values. We used a continuous assay to measure a K_m_ value of 290 μM for ATP (Fig 2C). This value is significantly higher than those measured for other tyrosine kinases; in a parallel experiment, we determined K_m_ (ATP) for human Abl to be 32 μM. When we measured the K_m_ (ATP) for MbAbl2 in the presence of Mn^2+^ rather than Mg^2+^, we obtained a value of 29 μM (S2A Fig). Next, we compared MbAbl2 activity towards synthetic peptides containing recognition motifs for various mammalian TKs (Abl, Src, insulin and EGF receptors) [22,24], as well as Kemptide, a prototypical substrate for the Ser/Thr kinase PKA [23]. MbAbl2 exhibited highest activity toward the Abl substrate, lower activity toward the Src and IR peptides, and was inactive against the EGFR and PKA substrates (Fig 2D). Thus, the substrate preference of the Abl catalytic domain appears to have been established early in the evolution of this family.

**Fig 3:**
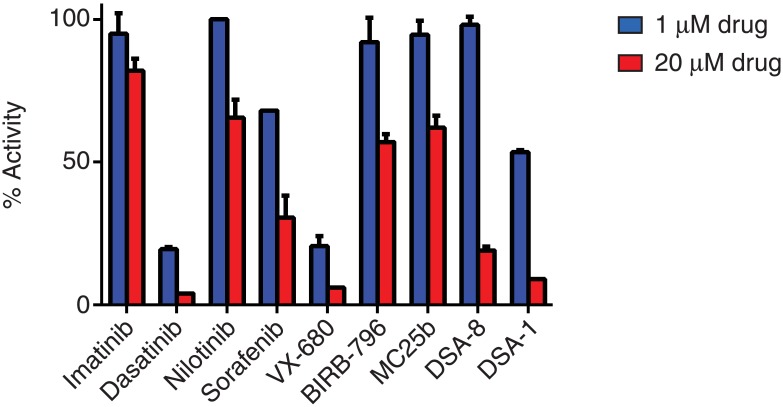
Effect of kinase inhibitors on MbAbl2. MbAbl2 activity was measured in the presence of 1 μM and 20 μM concentrations of the indicated inhibitors. Activity was measured using the phosphocellulose binding assay with Abl peptide substrate at a concentration of 125 μM. The concentration of ATP was 200 μM. Error bars show standard deviations.

The conformational states of c-Abl are differentially affected by small molecule kinase inhibitors. To gain insight into the conformational state of MbAbl2, we screened the enzyme against a panel of clinical and experimental kinase inhibitors at 1 μM and 20 μM (Fig 3). Imatinib (Gleevec), which selectively binds to the inactive, unphosphorylated (at Tyr412) form of c-Abl [27], gave only modest inhibition, and only at the higher concentration. We confirmed the effectiveness of imatinib in our assays using human Abl (IC_50_ = 39 nM; S3 Fig). Nilotinib, which also binds to the inactive form of Abl [30,31], was similarly effective only at 20 μM. Dasatinib is a dual Bcr-Abl/Src inhibitor that is more potent than imatinib against Bcr-Abl, and is also active against imatinib-resistant mutants [30,31]. Dasatinib inhibited MbAbl2, consistent with its ability to recognize multiple conformational states of Abl. VX-680, which recognizes the active conformation of c-Abl [32], was also effective against MbAbl2. These results suggest that MbAbl2 adopts a conformation that resembles the activated, DFG-in state of Abl with an extended activation loop. Sorafenib, a multikinase inhibitor of VEGFR, PDGFR, and Raf [33], gave partial inhibition of MbAbl2. The p38-alpha MAP kinase inhibitor BIRB-796 [34] and the Src-specific macrocycle MC25a [35] were inactive at 1 μM, but showed partial inhibition at 20 μM.

We tested two additional compounds based on the chemical scaffold of imatinib (DSA1 and DSA8) that were active against imatinib-resistant forms of Bcr-Abl containing mutations in the P-loop of the kinase domain [36]. These P-loop mutations (Y272H and E274V) occur in residues that form a deep pocket into which the hydrophobic portion of imatinib binds. MbAbl2 contains amino acid substitutions (relative to Abl) that could disrupt this pocket (R247 and D322), potentially explaining the loss of affinity towards imatinib. At 1 μM, DSA1 gave only partial inhibition against MbAbl2, while DSA8 was ineffective; both compounds showed inhibition at 20μM (Fig 3). Because DSA1 and DSA8 (like imatinib) require the inactive conformation of Abl for binding, these results are consistent with the idea that the lack of inhibition by imatinib is due to the ability of MbAbl2 to adopt an active-like conformation, rather than specific changes in the P-loop.

**Fig 4:**
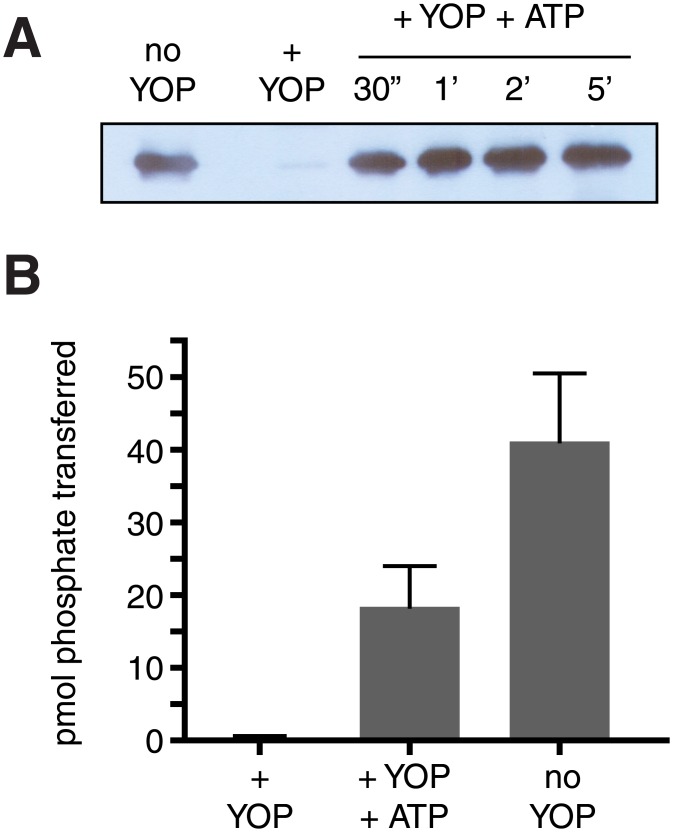
Regulation of MbAbl2 by autophosphorylation. (A) Purified MbAbl2 (200 μl of 825 nM) was incubated with immobilized GST-YOP tyrosine phosphatase (200 μl) for 45 minutes at room temperature, or left untreated (no YOP). Following removal of GST-YOP by centrifugation, autophosphorylation was initiated by the addition of ATP (0.5 mM) and MgCl_2_ (20 mM). After various reaction times (at room temperature), aliquots were removed and immediately quenched by addition of Laemmli sample buffer and boiling. The samples were analyzed by SDS-PAGE and anti-pTyr Western blotting; equal amounts of MbAbl2 (14 pmol) were analyzed in each lane. (B) YOP treatment and autophosphorylation were carried out as in panel (A). After 30 min. of autophosphorylation, MbAbl2 activity was measured using the phosphocellulose paper binding assay with Abl peptide (740 μM). Kinase reactions proceeded for 10 minutes at 30°C. Error bars show standard deviations.

Mammalian forms of Abl undergo intermolecular autophosphorylation at Tyr412 within the activation loop in the catalytic domain [1,27]. Phosphorylation at Tyr412 triggers a conformational change that increases enzymatic activity. To test whether this mode of regulation was present in ancestral forms of Abl, we treated purified MbAbl2 with the Yersinia tyrosine phosphatase (YOP). This treatment removed background tyrosine phosphorylation on MbAbl2 that occurred during expression and purification from insect cells (Fig 4A). YOP also decreased the activity of MbAbl2 to undetectable levels (Fig 4B). After removal of immobilized YOP, we incubated MbAbl2 with ATP and Mg^2+^ under conditions that promote c-Abl autophosphorylation. MbAbl2 rapidly autophosphorylated under these conditions, as assessed by anti-pTyr western blotting (Fig 4A). At the same time, we observed a partial recovery of enzymatic activity (Fig 4B). We measured the K_m_ (ATP) for dephosphorylated and rephosphorylated MbAbl2 (S2B Fig). It was 250 μM, similar to the value for the enzyme without these treatments (Fig 2C). These results suggest that autophosphorylation evolved early as a mechanism for Abl kinase regulation. We reached similar conclusions for premetazoan forms of Src [17–19].

**Fig 5:**
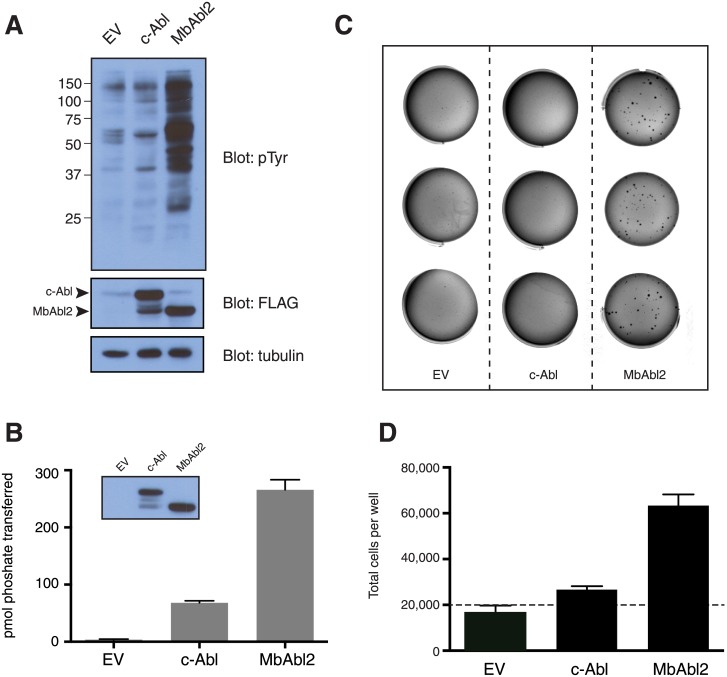
Abl activity in mammalian cells. (A) Lysates from NIH3T3 cells stably expressing Abl, MbAbl2, or empty vector (EV) (100 μg) were separated by SDS-PAGE and analyzed by anti-pTyr Western blotting. The membrane was stripped and reprobed with anti-Flag and tubulin antibodies to ensure equal loading of Abl and MbAbl2. The figure is representative of three independent experiments. (B) Lysates from NIH3T3 cells expressing Abl-Flag or MbAbl2-Flag (1 mg total protein) were subjected to immunoprecipitation reactions with anti-Flag affinity resin. Duplicate samples of the precipitated proteins were used in an *in vitro* kinase reaction with Abl peptide and [γ -^32^P]-ATP. Error bars show standard deviations. A separate sample was analyzed by Western blotting with anti-Flag antibody (inset). (C) Colony formation assay. NIH3T3 cells expressing Abl, MbAbl2, or vector control were sorted for equal GFP expression. Cells (1000 per well) were resuspended in low melting point agarose prepared in complete media. Wells were photographed after 28 days. This experiment was repeated three times with similar results. (D) Anchorage-independent growth assay. NIH3T3 cells expressing Abl, MbAbl2, or vector control (20,000 per well; dashed line) were resuspended in complete media and added to ultra low attachment plates. Cells were counted after 6 days of growth. This experiment was performed three times with similar results. Error bars show standard deviations.

Our results on purified MbAbl2 suggested that the enzyme had high constitutive activity. To test this in a cellular context, we used a retroviral expression strategy to generate an NIH3T3 cell line expressing full-length MbAbl2. We produced an analogous NIH3T3 cell line for mouse c-Abl. This construct consisted of the murine c-Abl N-terminus, SH3, SH2, and kinase domains (residues 1–660). Thus, the c-Abl construct had a similar domain architecture as MbAbl2, and it corresponded to the c-Abl fragment crystallized by Kuriyan and coworkers, which was shown to be in its autoinhibited conformation [2]. Both constructs contained a C-terminal Flag tag to avoid interference with the N-terminal myristoylation of c-Abl. We showed that the activity of Flag-tagged Abl was comparable to that of full-length c-Abl (S4 Fig). The retroviruses encoded GFP (on a separate polypeptide) together with Abl or MbAbl2; we monitored infections using GFP fluorescence, and we obtained homogeneous populations of NIH3T3 cells with similar levels of expression by preparative FACS. We first assessed the activities of c-Abl and MbAbl2 by measuring overall tyrosine phosphorylation of NIH3T3 cellular proteins. The activity of c-Abl in this assay was only marginally above background in this experiment (Fig 5A); this result recapitulates previous findings showing that normal c-Abl proteins were not tyrosine phosphorylated, even with expression levels 10-fold higher than normal [37]. In contrast, MbAbl2 showed strong phosphorylation of multiple cellular proteins in this experiment (Fig 5A). We immunoprecipitated the Flag-tagged kinases from NIH3T3 cells and showed that MbAbl2 had higher activity than Abl toward a synthetic peptide substrate (Fig 5B).

A key measure of cellular transformation is the ability of oncogenes to induce anchorage-independent growth. We measured the ability of c-Abl and MbAbl2 to transform NIH3T3 cells by comparing their ability to induce colony formation in soft agar. MbAbl2 expression caused colony formation (54.3 ± 4.7 colonies per plate), whereas c-Abl did not (Fig 5C). Next, we measured the growth of NIH3T3 cells expressing c-Abl or MbAbl2 on ultralow attachment plates. The initial number of cells seeded in each well was 20,000. After 6 days, vector-transformed control NIH3T3 cells showed a small decrease in cell numbers, and Abl-expressing cells showed a small increase. In contrast, MbAbl2 strongly promoted growth in the absence of attachment (Fig 5D). These results show that the transforming ability of MbAbl2 is significantly higher than that of normal c-Abl, and are consistent with the constitutive activity observed for the purified MbAbl2 kinase.

## Discussion

The genome of the unicellular choanoflagellate *M*. *brevicollis* encodes numerous receptor and nonreceptor tyrosine kinases [8]. Many of the nonreceptor tyrosine kinases contain domain combinations that have not been observed in multicellular animals [7,9]. These arrangements of domains may have evolved originally to facilitate recognition of substrates, followed by the development of stringent catalytic regulation later in the metazoan lineage [38]. Some of the *M*. *brevicollis* nonreceptor tyrosine kinases (Abl, Src, Csk, Tec) belong to recognizable metazoan families. Nonreceptor tyrosine kinases containing the SH3-SH2-kinase architecture have also been observed in earlier-branching sister groups to choanoflagellates: filastereans, ichthyosporeans, and a corallochytrean [10,11,39]. Abl family kinases are present in the filastereans *C*. *owczarzaki* and *M*. *vibrans* and in the ichthyosporeans *A*. *whisleri*, *P*. *gemmata*, and *A*. *parasticium* [11]. The normal cellular functions of Abl kinases in premetazoan lineages, however, remain enigmatic. The combination of the SH2 and Abl kinase catalytic domains, present in all analyzed examples of Abl, may have been especially important for the development of complex signaling circuits in early kinase evolution [40]. Comparative studies of premetazoan and metazoan TKs can illuminate the evolutionary development of kinase regulatory mechanisms.

The cellular activity of Abl tyrosine kinase is tightly controlled by multiple autoinhibitory mechanisms [2]. Similar to Src family TKs, the SH3 and SH2 domains make intramolecular interactions on the distal side of the catalytic domain (opposite the peptide binding site). The SH3 domain binds to a linker peptide that connects the SH2 and kinase domains, while the SH2 domain forms a close protein-protein interface with the C-lobe of the catalytic domain. Uniquely to Abl, the N-terminal myristoyl moiety binds to a deep hydrophobic pocket in the catalytic domain. This interaction is important for autoinhibition. The N-terminal cap segment supports the SH3 and SH2 domains and helps maintain the autoinhibitory conformation. The importance of the myristate and N-terminal cap for Abl regulation is highlighted by early studies in which N-terminal deletions activate the transforming ability of Abl [37]. In CML, autoinhibition is disrupted by the fusion of Bcr N-terminal to the SH3 domain, leading to removal of the cap region [3].

We studied the biochemical properties of MbAbl2 using the purified enzyme. The *M*. *brevicollis* enzyme, like mammalian Abl, recognizes peptide sequences containing the IYAAP sequence (Fig 1C). Like c-Abl, MbAbl2 is activated by autophosphorylation (Fig 4). MbAbl2 demonstrated an unusually high K_m_ toward ATP. This may be due to a Val residue in the predicted adenine-binding pocket of MbAbl2; Met is commonly found at that position in kinases, and the Met residue makes H-bonds with the nitrogen atoms in the adenine ring of ATP. Our data indicate that the conformation of *M*. *brevicollis* MbAbl2 resembles that of activated forms of mammalian Abl. In a survey of small molecule kinase inhibitors, compounds that recognize activated forms of Abl (dasatinib and VX-680) showed inhibition at 1 μM, while those that interact with the inactive form (imatinib, nilotinib) did not (Fig 3). There are numerous sequence differences between Abl and MbAbl2 that could underlie the high constitutive activity of the *M*. *brevicollis* enzyme. One such difference occurs within the activation loop. His396 in Bcr-Abl is often mutated to proline or arginine in patients with early-phase CML, and causes the DFG motif to adopt an activated conformation that places the Asp residue oriented inward toward the kinase domain [31]. This DFG-in orientation is conserved among most activated tyrosine kinases as it promotes a salt bridge between the Asp residue and a Mg^2+^ ion needed for catalysis. MbAbl2 contains an Arg residue at the position corresponding to His396 of Bcr-Abl, which may contribute to constitutive activation. Another mutation occurs in the ATP binding site at the “gatekeeper” position, also a site of frequent mutation in imatinib-resistant CML. The T315I mutation in Bcr-Abl renders the enzyme resistant to imatinib and increases activity [41]. MbAbl2 has a Gln residue at this position, rather than the Thr that is present in Abl and most tyrosine kinases. The Gln residue at this position of MbAbl2 may also contribute to the high specific activity of this enzyme [42].

When comparable c-Abl and MbAbl2 constructs were expressed at similar levels in mammalian cells, we found that the activity of MbAbl2 was far higher (Fig 5). Moreover, some of the cellular proteins phosphorylated by MbAbl2 were presumably physiological substrates of Abl, since expression of MbAbl2 induced cellular transformation (Fig 5C and 5D), as has previously been shown for activated forms of mammalian Abl kinases [37]. The presence of the SH2 domain in MbAbl2 would permit processive phosphorylation [43–45], although in preliminary experiments we did not observe enhanced phosphorylation of peptides containing SH2 ligand sequences (data not shown). The identification of MbAbl2 substrates in mammalian and *M*. *brevicollis* cells is ongoing.

Our data are consistent with a model in which the regulatory elements for Abl kinases evolved later in the metazoan lineage. We and others have postulated similar processes for the Src and Pak proto-oncogenes [17,38,46]. A caveat is that the *M*. *brevicollis* kinome includes an additional Abl-like kinase, MbAbl1 [7]. This kinase is predicted to contain an N-terminal Gly residue that could potentially be myristoylated. Whether this kinase (or any other *M*. *brevicollis* protein) is myristoylated *in vivo* is currently unknown, and the possibility that MbAbl1 might be regulated by autoinhibition has not been explored. Another elaboration during metazoan evolution was the extended C-terminus, containing binding motifs for F-actin and for DNA. Thus, it is clear that additional functionality was appended to Abl kinase as part of the transition to multicellularity.

## Supporting Information

S1 Fig Amino acid alignment of c-Abl (murine c-Abl1b) and MbAbl2. The SH3, SH2, and kinase catalytic domains are highlighted and colored as in Fig 1. The major autophosphorylation site in c-Abl (Tyr412) is indicated with a red arrow.
(EPS)

S2 Fig Additional K_m_ (ATP) measurements. (A) MbAbl2 initial rates were measured at varying concentrations of ATP in the presence of MnCl_2_ using the continuous spectrophotometric assay. The fit to a hyperbolic substrate vs. velocity curve is shown. (B) Initial rates for YOP-treated and rephosphorylated MbAbl2 were measured at varying concentrations of ATP in the presence of MgCl_2_ using the continuous spectrophotometric assay.
(EPS)

S3 Fig Effectiveness of imatinib against Abl. (A) Human Abl activity in the presence of varying concentrations of imatinib was measured using the spectrophotometric assay. The inhibition curve was fit using GraphPad Prism.
(EPS)

S4 Fig Effect of the Flag tag. Lysates from NIH3T3 cells expressing Flag-Abl or Flag-MbAbl2 (2.7 mg total protein) were subjected to immunoprecipitation reactions with anti-Flag affinity resin. (A) Duplicate samples of the precipitated proteins were used in an *in vitro* kinase reaction with Abl peptide and [γ-^32^P]-ATP. Error bars show standard deviations. (B) A separate sample was analyzed by Western blotting with anti-Flag antibody.
(EPS)
